# Pyoderma Gangrenosum in an African American Male Initially Presenting as Sepsis

**DOI:** 10.7759/cureus.21592

**Published:** 2022-01-25

**Authors:** Carly E Wallace, Amit Sharma

**Affiliations:** 1 College of Osteopathic Medicine, Lake Erie College of Osteopathic Medicine, Bradenton, USA; 2 College of Osteopathic Medicine, Lake Erie College of Osteopathic Medicine, Elmira, USA

**Keywords:** pathergy, soft tissue infection, neutrophilic dermatosis, sepsis, pyoderma gangrenosum

## Abstract

Pyoderma gangrenosum (PG) is a rare, ulcerating, rapidly developing neutrophilic dermatosis that is often challenging to diagnose and treat. We present the case of a 47-year-old African American male who presented with a painful left anterior shin ulcer, fever, leukocytosis, and tachycardia. The patient had a similar lesion seven years prior that had since healed, with no other medical conditions. Sepsis secondary to a soft tissue infection was initially suspected; however, given the patient’s history of pathergy, rapid progression of the lesion, skin examination, and sterile wound culture, PG was diagnosed. The patient improved in response to corticosteroid therapy. A brief overview of the disease presentation, diagnosis, and treatment is provided.

## Introduction

Pyoderma gangrenosum (PG) is an uncommon neutrophilic dermatosis characterized by rapidly progressive skin ulceration. These painful ulcers appear most frequently on the lower extremities. The etiology of this disease is poorly understood but is related to neutrophil dysfunction with the involvement of lymphocytes and cytokines [1]. PG is associated with other immune-mediated diseases, including inflammatory bowel disease and rheumatoid arthritis. The diagnosis of PG can be deduced from a patient’s history, clinical appearance, histopathology, presence of associated diseases, and response to therapy. Topical therapy can be used for lesions that are <2 cm^2^, while systemic corticosteroids are the first-line treatment for more extensive cases [2].

## Case presentation

A 47-year-old African American male with Fitzpatrick skin type V presented to the hospital with a painful ulcer on the anterior aspect of his left shin. Initial insult had occurred five days prior following minor trauma. The lesion subsequently increased in size and became purulent, erythematous, and ulcerating. A similar episode had occurred seven years ago when the patient developed a lesion on the right shin that was treated with surgical debridement and a skin graft. The patient had no other medical history and was not on any medications.

**Figure 1:**
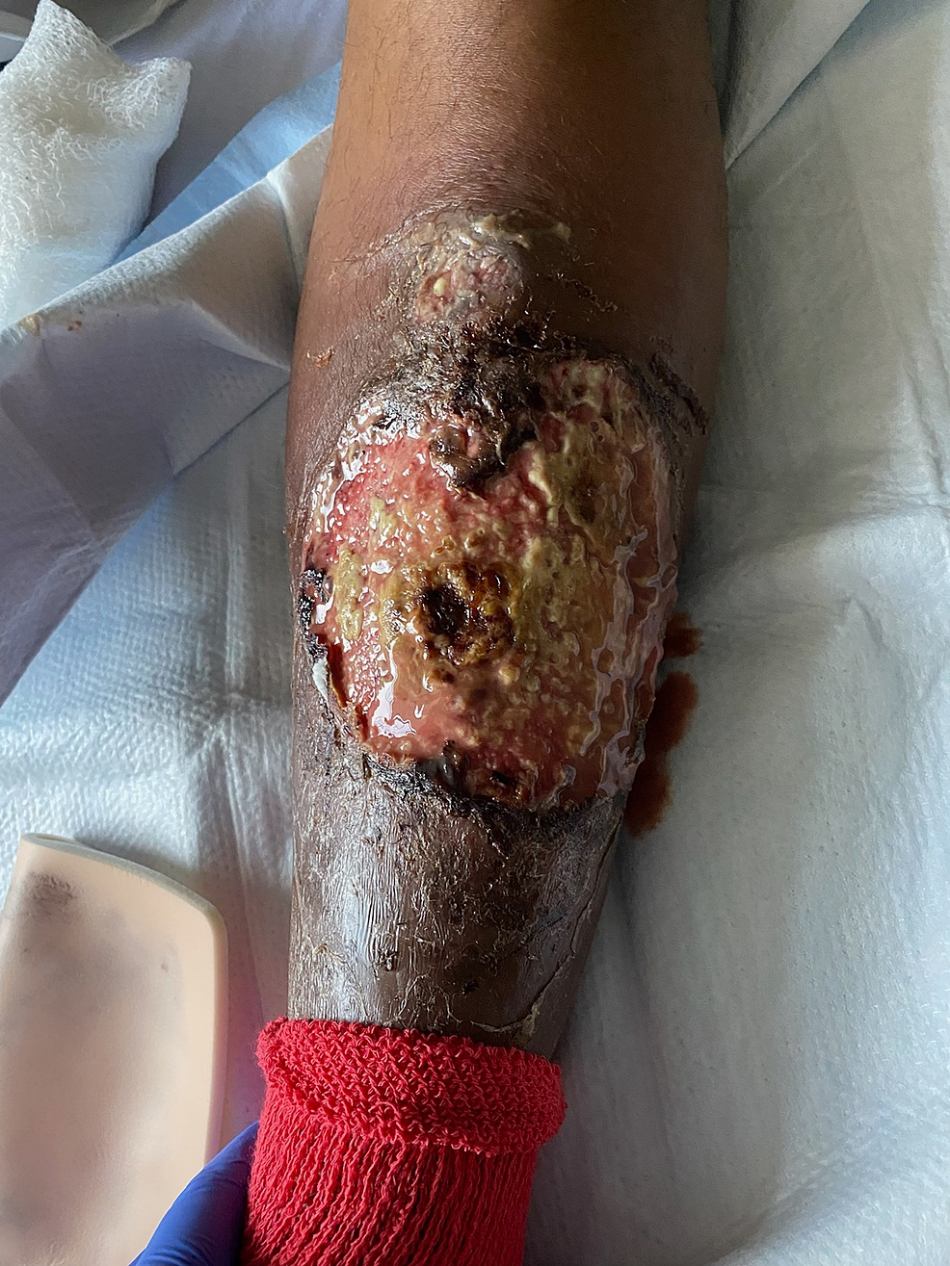
Cutaneous examination revealed extensive ulceration of the shin with violaceous undermined borders and purulent discharge.

Vital signs at admission were significant for a temperature of 38.8°C and a heart rate of 118 beats/minute. Physical examination revealed a 16 × 12 × 0.2 cm wound that was 70% dry, exposed subcutaneous tissue and 30% moist, red-pink-yellow subcutaneous tissue (Figure 1). There were violaceous undermining borders, warmth, and clear purulent discharge. Extreme tenderness to palpation was present around the wound and wound edge. There was no crepitus on palpation, ecchymosis, edema, or lymphadenopathy.

The laboratory blood work was significant for the following: WBC, 15,300/mm^3^; CRP, 12.4 mg/dL; ESR, 87 mm/hour; complement total blood test (CH50), >95.0 U/m: and complement component 3 (C3), 216 mg/dL. Lactic acid and complement component 4 (C4) were normal. COVID-19 testing was negative. X-ray of the left tibia/fibula showed soft tissue edema corresponding to the wound, but no signs of osteomyelitis. Wound and blood cultures did not show any growth. Further testing revealed positive ANA and anti-Ro/SSA. Double-stranded DNA, rheumatoid factor, Jo 1, Scl-70, cyclic citrullinated peptide, anti-RNP, anti-Smith, and anti-La/SSB were all negative.

Sepsis criteria were initially met, defined as two or more of the systemic inflammatory response syndrome criteria plus a suspected source of infection [3]. The patient met three of the four of these criteria upon initial evaluation in the hospital, as he had a fever, tachycardia, and leukocytosis. Soft tissue infection was initially suspected, prompting treatment with IV vancomycin and piperacillin/tazobactam.

Given the patient’s history of pathergy, rapid progression of the lesion, skin examination, and sterile wound culture, the patient was diagnosed with PG. He was started on a treatment regimen of IV methylprednisolone. The wound began to show signs of improvement, decreasing in size to 14.5 × 12.5 × 0.1 cm. Vital signs stabilized within one day of hospitalization, and repeat ESR and CRP were normal.

**Figure 2:**
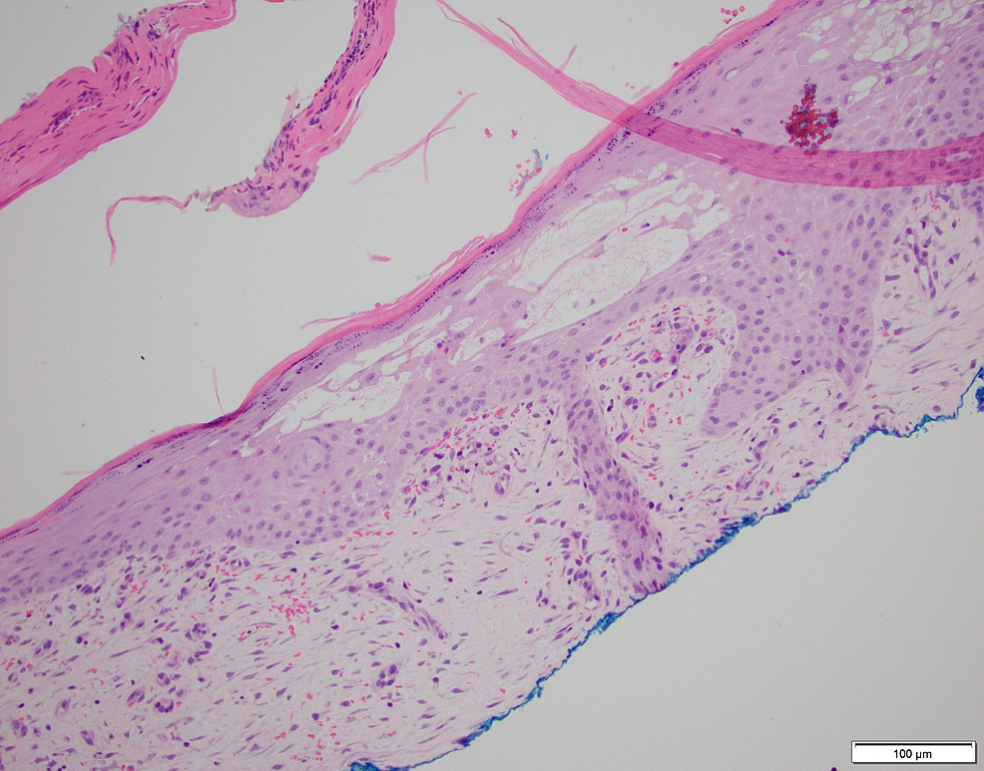
Biopsy demonstrated dermal edema, extravasated erythrocytes, and a patchy superficial perivascular chronic inflammation. There is no neutrophilic infiltrate seen.

The patient remained in the hospital for four days and was discharged on oral prednisone. Shave biopsy was taken two weeks later and showed dermal edema, extravasated erythrocytes, and patchy superficial perivascular chronic inflammation (Figure 2). No organisms were seen. Following completion of treatment, the wound had resolved by two months.

## Discussion

PG is a noninfectious autoinflammatory dermatosis characterized by painful skin ulceration with violaceous undermining borders. It has an annual incidence of 3-10 cases per million population with a similar prevalence among African American and White patients. Most patients with PG are 50 years or older, with a median age of 59 and a peak age of onset of 44 years. There is a slight female preponderance, with women comprising 59% of cases [4-6].

PG is highly associated with systemic diseases such as inflammatory bowel disease, rheumatoid arthritis, and hematological disorders [1]. In our case, the patient did not have any diagnosed medical conditions, but there was a history of possible PG in the contralateral lower extremity several years prior. The patient’s ANA and anti-Ro/SSA were positive; however, he did not complain of any symptoms that would suggest an underlying autoimmune disease.

Differential diagnosis of PG includes vascular occlusion or stasis, vasculitis, cutaneous involvement by a malignant process, primary cutaneous infection, drug-induced tissue injury, and other inflammatory disorders [7]. While a soft tissue infection was suspected when the patient was initially admitted to the hospital, wound cultures did not show any bacterial growth. Furthermore, the patient’s lesion responded to treatment with corticosteroid therapy, supporting the diagnosis of PG.

In the past, PG has been described as a diagnosis of exclusion [7]. However, recent diagnostic criteria have been proposed, including a combination of ulcer edge biopsy, patient history, clinical examination, and response to treatment [8,9]. In the present case, the patient’s history of pathergy, rapid ulceration of the lesion, examination findings, and resolution with corticosteroid therapy were all consistent with the proposed criteria. However, the biopsy findings did not show the characteristic features of PG. Biopsy of the ulcer edge demonstrating a neutrophilic infiltrate with the exclusion of infection is consistent with a diagnosis of PG. In our case, the biopsy did not show a dense neutrophilic infiltrate but rather dermal edema and numerous lymphocytes, indicative of chronic inflammation. There are two possibilities as to why this occurred: the biopsy was not taken from the ulcer edge or the lesion was partially resolved at the time of biopsy, as it was obtained two weeks after the start of treatment.

A multicenter study found that patients with PG were predominantly treated with systemic therapy, while 17% were treated with topical therapy alone. The most used systemic drugs include steroids, cyclosporine, dapsone, and infliximab. In most cases, systemic therapy utilized one drug, with 25% requiring a combination of two treatments [9,10]. In this case, since the lesion was too large to adequately treat with topical therapy alone, oral prednisone was used for systemic therapy. Other aspects of treatment include wound care, avoidance of triggers and trauma, analgesia, lifestyle modification, prevention of superimposed infection, and compression therapy [11]. In our patient, an antibiotic ointment was utilized to avoid infection.

## Conclusions

This case emphasizes the importance of obtaining detailed patient history and wound culture in patients initially suspected of having a soft tissue infection. The diagnosis of PG is often delayed or missed; thus, it should be considered as a differential diagnosis in patients presenting with rapid skin ulceration. This case was unique in that the patient initially met sepsis criteria and was treated accordingly. However, when the wound and blood cultures did not show any growth, we took a closer look at the patient’s history, which ultimately led us to the diagnosis of PG.
